# Zinc‐Modified Sulfonated Polyetheretherketone Surface with Immunomodulatory Function for Guiding Cell Fate and Bone Regeneration

**DOI:** 10.1002/advs.201800749

**Published:** 2018-08-07

**Authors:** Wei Liu, Jinhua Li, Mengqi Cheng, Qiaojie Wang, Kelvin W. K. Yeung, Paul K. Chu, Xianlong Zhang

**Affiliations:** ^1^ Department of Orthopaedics Shanghai Jiao Tong University Affiliated Sixth People's Hospital Shanghai Jiao Tong University Shanghai 200233 China; ^2^ Department of Orthopaedics and Traumatology Li Ka Shing Faculty of Medicine The University of Hong Kong Pokfulam Hong Kong 999077 China; ^3^ Department of Physics and Department of Materials Science and Engineering City University of Hong Kong Tat Chee Avenue Kowloon Hong Kong 999077 China

**Keywords:** bone formation, immunomodulation, macrophages, stem cells, zinc

## Abstract

The cytokines released by immune cells are considered important factors to induce bone tissue regeneration. However, the pathway of those bone‐targeting macrophage cytokines induced by biomaterial surface under tissue microenvironment is rarely reported. In this study, the immunomodulatory capability of zinc ions on macrophage polarization and its effects on osteogenic differentiation are investigated. Hence, a layer of zinc ions are incorporated on sulfonated polyetheretherketone (SPEEK) biomaterials by using a customized magnetron sputtering technique. The results reveal that the microenvironment on Zn‐coated SPEEK can modulate nonactivated macrophage polarization to an anti‐inflammatory phenotype and induce the secretion of anti‐inflammatory and osteogenic cytokines. The osteogenic differentiation capability of bone marrow stromal cells (BMSCs) is therefore enhanced, leading to improved osteointegration between the zinc‐coated SPEEK and bone tissue. This study verifies that zinc ion is a promising additive in the osteoimmunomodulation process and provides knowledge that may pave the way to develop the next generation of immunomodulatory biomaterials.

## 1 Introduction

In recent years, the progresses made in immunology and the deepened understanding of bone remolding have led to the emergence of a new word, “osteoimmunology,” indicating the close relation between the immune system and skeletal system.^1^ The immune cells in bone can influence bone remolding and resorption because of the set of signaling molecules, cytokines, and receptors they share with the skeletal system.[[qv: 1b,2]] Regarding bone regeneration materials, the traditional strategy involves targeting osteoblastic lineage cells and fabricating direct osteogenic biomaterials to stimulate osteogenesis.^3^ While some inconsistencies were observed between in vitro and in vivo outcomes for these direct osteogenic biomaterials, further studies demonstrated that the immune reactions triggered by biomaterials may be responsible for this phenomenon.^4^ These findings demonstrated that advanced strategies for bone regeneration materials should focus on not only direct osteogenesis but also the development of immunomodulatory materials to generate a favorable immune environment and achieve satisfactory osteointegration.

When biomaterials are implanted in vivo, a series of biological reactions are triggered. However, the first host response to implants is an innate immune response (foreign body reaction).^5^ Several types of immune cells are sequentially recruited to the implant site and trigger immune reactions. However, an acute or severe immune response will impair the process of osteogenesis and result in encapsulation of the implant. In contrast, a suitable anti‐inflammatory immune response induced by the implant is beneficial for bone repair and angiogenesis.^6^ Thus, an immunomodulatory biomaterial that can regulate immune cells to secrete osteogenic cytokines and can maintain an optimal immune microenvironment for bone repair should hold promise in clinical applications.^7^

To design an immunomodulatory implant, we must clarify the dominant immune cells in the host immune response to biomaterials. Macrophages, as the frontline of tissue–implant interactions, are one of most important effector cells for the foreign body reaction. Macrophages were traditionally considered an “evil” that secretes inflammatory cytokines and is detrimental to osteogenesis. However, subsequent studies have demonstrated that macrophages are a “double‐edged sword.”^8^ Specifically, macrophages are plastic and dynamic: when stimulated by different signals, macrophages can polarize to classically activated inflammatory macrophages (M1) with CCR7 as typical surface markers or alternatively activated inflammatory macrophages (M2) with CD206 as typical surface markers.^9^ Generally, macrophages turn to M1 phenotype when infection or “danger” happens and then secrete proinflammatory mediators such as TNF‐α or IL‐6, which will impair osteogenesis and would heal when an excess amount is released.[[qv: 9c,10]] In contrast, M2 phenotype macrophages produce IL‐4 or IL‐10 to provide an anti‐inflammatory microenvironment facilitating tissue healing.^11^

Recently, several studies have demonstrated that the immune microenvironment generated by biomaterials can be modulated by their surface microstructure, wettability, particle size, porosity, and released ions.[[qv: 1a,12]] Regarding the active ions released, studies have verified that magnesium (Mg), strontium (Sr), and copper (Cu) can suppress inflammatory cytokines secreted by macrophages and promote osteogenesis.^13^ Zinc, an essential trace element constituting some key enzymes and transcription factors, was reported to be indispensable for the development of the immune system, and suitable amounts of zinc can enhance the expression of anti‐inflammatory cytokines and maintain an anti‐inflammatory environment.^14^ However, no relevant study has focused on the osteoimmunomodulatory effect of zinc on osteogenic differentiation or osteointegration. In this study, we investigated the osteoimmunomodulatory ability of zinc by coating zinc on sulfonated polyetheretherketone (SPEEK) and culturing macrophages on it and performed further in vitro and in vivo experiments on osteogenesis in the immune environment generated by Zn‐coated SPEEK. Our study elucidated the further application of zinc in immunomodulatory and bone regeneration biomaterials.

## 2 Results

### 2.1 Characteristics of Zn‐Coated SPEEK

**Figure 1:**
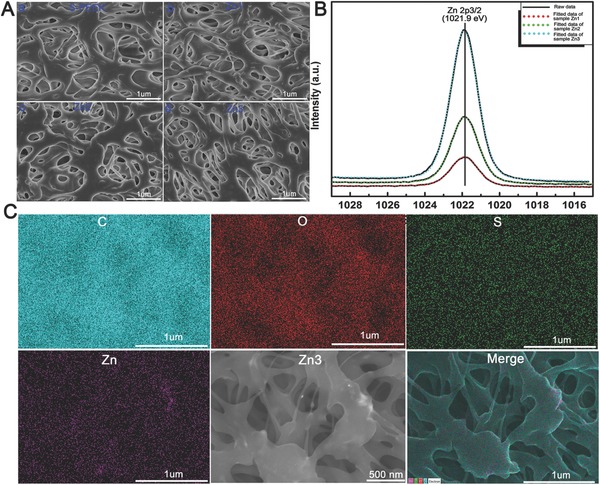
Characterization of different samples. A) Scanning electron images of SPEEK, Zn1, Zn2, and Zn3. B) XPS results of Zn1, Zn2, and Zn3. C) EDS mapping for the major elements of Zn coated SPEEK (Zn3).

A 3D porous Zn (three concentration gradients) coating on PEEK was fabricated by sequential sulfonation and magnetron sputtering. According to scanning electron microscopy (SEM) analysis, a stratified porous surface structure was formed on the surface of PEEK after sulfonation treatment (**Figure** **1**A). The Zn‐coated SPEEK showed similar surface topography to SPEEK, which indicated that incorporating zinc on the surface by magnetron sputtering exerted little influence on the surface structure. The binding energy of the Zn 2p_3/2_ X‐ray photoelectron spectroscopy (XPS) peak at 1021.9 eV indicated the presence of zinc in the oxide form (Figure 1B). Energy‐dispersive X‐ray spectrometry (EDS) mapping (Figure 1C) and XPS results revealed that zinc was incorporated successfully in the coating and distributed homogeneously. Determined by XPS, the contents of Zn for Zn1‐SPEEK, Zn2‐SPEEK, and Zn3‐SPEEK were 3.57, 6.73, 15.97 at%, respectively (Table S1, Supporting Information). The bonding strengths of Zn coating for Zn‐coated SPEEK were also examined and the results were 5.2, 5.2, and 5.4 MPa for Zn1‐SPEEK, Zn2‐SPEEK, and Zn3‐SPEEK, respectively (Table S1, Supporting Information). The contact angles (CAs) of the samples were also determined, and the contact angles for PEEK, SPEEK, Zn1‐SPEEK, Zn2‐SPEEK, and Zn3‐SPEEK were 103.66 ± 3.51, 98.00 ± 1.00, 96.67 ± 2.08, 94.67 ± 1.05, and 93.67 ± 1.52, respectively. The contact angle of PEEK was higher than those of other groups (*p* < 0.05, Figure S1, Supporting Information). Sulfonation treatment decreased the contact angles of the surface to some degree, but no significant difference was found between SPEEK and Zn‐coated SPEEK (Figure S1, Supporting Information). To determine pH values and zinc release concentrations, immersion tests were performed by immersing samples in Dulbecco's modified Eagle's medium (DMEM) at 37 °C for 1, 4, 7, and 14 d. Figure S2 (Supporting Information) showed similar pH (≈8) curves of SPEEK and Zn‐coated SPEEK, suggesting that the acidic substances on the surface were cleared and that Zn incorporation exerted minimal effects on pH. Figure S3 (Supporting Information) shows that the zinc release was relatively high on day 1 and that the zinc release rate then slowed down in the following days, with a highest zinc concentration of ≈160 µg L^−1^ (0.16 ppm).

### 2.2 In Vitro Biocompatibility of Zn‐Coated SPEEK

**Figure 2:**
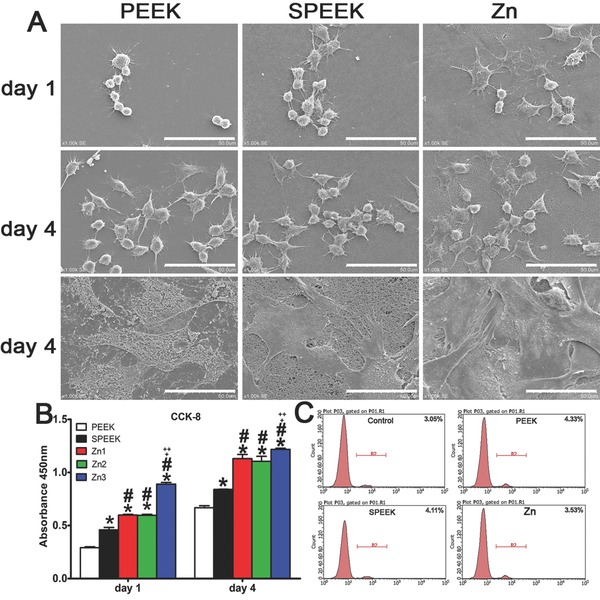
A) Scanning electron images of RAW264.7 and rBMSC cells on PEEK, SPEEK and Zn, bar: 50 µm. B) CCK8 results of RAW264.7 cultured on PEEK, SPEEK and Zn for 1 and 4 d. C) Percentage of dead cells culture on materials using PI staining determined by flow cytometry. (*, #, +, and ++ represent *p* < 0.05 when compared with PEEK, SPEEK, Zn1, Zn2 respectively). (*, #, +, and ++ represent *p* < 0.05 when compared with PEEK, SPEEK, Zn1, Zn2 respectively).

To study the in vitro biocompatibility of samples, both macrophage‐like RAW 264.7 cells and rat bone marrow mesenchymal stem cells (rBMSCs) were seeded on samples. SEM images showed that macrophages and rBMSCs adhered well on the modified surfaces and that there were more cells on Zn‐coated SPEEK (denoted “Zn” in the following figures) than on SPEEK. In addition, the cells seemed to spread flatter and more diversely on Zn‐coated SPEEK, especially the RAW 264.7 cells (**Figure** **2**A). The CCK‐8 assay was employed to evaluate the proliferation of macrophages on samples, and the results revealed that Zn3‐coated SPEEK afforded the highest cell viability (Figure 2B). Furthermore, the proportion of dead cells was calculated by staining macrophages with propidium iodide (PI) and then analyzing them by flow cytometry. Zn‐coated SPEEK exhibited a similar number of dead cells as the blank control surface but fewer dead cells than PEEK or SPEEK (Figure 2C). According to the SEM images and CCK‐8 and dead cell analyses, we concluded that among the materials tested, Zn3‐coated SPEEK possessed the best biocompatibility. Moreover, preliminary experiments on macrophage phenotype switching demonstrated that Zn3‐coated SPEEK had the strongest ability to induce macrophage polarization, but there was no significant difference among Zn1, Zn2, and Zn3‐coated SPEEK. Therefore, the subsequent in vitro and in vivo tests were performed by using Zn3‐coated SPEEK as Zn‐coated SPEEK (denoted “Zn” in the following figures).

### 2.3 Gene Expression of Macrophages Cultured on Samples

#### 2.3.1 Gene Expression Profile of Macrophages Analyzed by Microarray

**Figure 3:**
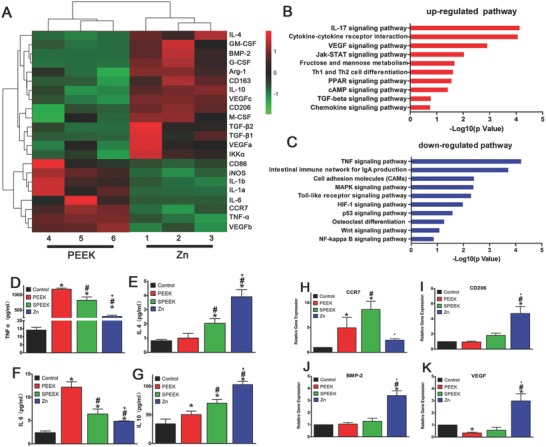
Gene expression analysis of RAW264.7 cultured on samples. A) Microarray heat map depicting the fold change of selected genes expression. B–C) Representative Top 10 upregulated or downregulated pathways analyzed by KEGG pathway method. D–G) Elisa results of TNF‐ɑ, IL‐4, IL‐6, and IL‐10 respectively. H–K) RT‐PCR results of CCR7, CD206, BMP‐2, and VEGF respectively. (*, #, and + represent *p* < 0.05 when compared with Control, PEEK, SPEEK, respectively).

RNA‐Seq was employed to detect the whole mRNA expression of macrophages cultured on samples for 4 d. **Figure** **3**A displays the heat map of selected genes (surface markers for different phenotype macrophages or cytokines) and depicts expression fold changes between PEEK and Zn‐coated SPEEK. Clearly, the expression levels of surface markers for M2 macrophages (CD206 and CD163) were enhanced on Zn‐coated SPEEK. In contrast, the CCR7 and iNOS genes, markers for M1 macrophages, were downregulated (Figure 3A). In addition, the expression of some osteogenic genes, such as BMP‐2, vascular endothelial growth factor (VEGF), and TGF‐β, increased when cells were cultured on Zn‐coated SPEEK. To further understand the signaling pathways involved in regulating macrophage phenotype switching and osteogenic gene expression, kyoto encyclopedia of genes and genomes (KEGG) pathway analysis was applied to determine the upregulated or downregulated pathways. Figure 3B,C shows ten representative upregulated and downregulated signaling pathways, respectively. Specifically, the NF‐κB signaling pathway, a M1 phenotype‐related pathway, was slightly downregulated. Moreover, the Jak‐STAT signaling pathway, a key pathway regulating M2 polarization, was significantly upregulated, indicating the switch in the macrophage phenotype to M2. Regarding the pathways that may lead to the expression of osteogenic cytokines, the cytokine–cytokine receptor interaction, VEGF, and TGF‐β signaling pathways were upregulated (Figure 3B). At the same time, the TNF‐α signaling pathway was significantly downregulated (Figure 3C). In brief, the microarray assay data suggested that Zn‐coated PEEK can induce a change in the gene expression profile of macrophages to the M2 phenotype profile while promoting the secretion a series of cytokines that are beneficial for bone regeneration and angiogenesis.

#### 2.3.2 Enzyme‐Linked Immunosorbent Assay (ELISA) of Inflammation‐Related Cytokines

To further validate the representative cytokines secreted by M1 and M2 macrophages, ELISA was employed to determine the concentrations of TNF‐α, IL‐6, IL‐4, and IL‐10. The results are presented in Figure 3D–G. Macrophages on Zn‐coated SPEEK secreted the highest amounts of the anti‐inflammatory cytokine IL‐4 and IL‐10, which are mainly produced by M2 macrophages. In contrast, the expression levels of two inflammatory cytokines, TNF‐α and IL‐6, were the lowest on Zn‐coated SPEEK. The findings of ELISA were highly consistent with those of the microarray assay.

#### 2.3.3 RT‐PCR of Surface Markers and Osteogenic Cytokines

To further confirm the ability of Zn‐coated PEEK to exert immunomodulatory effects and induce osteogenic cytokine production, we selected some representative genes to determine their fold changes by real‐time polymerase chain reaction (RT‐PCR). First, the typical M2 macrophage marker CD206 was upregulated on Zn‐coated PEEK (Figure 3I), suggesting a higher proportion of M2 macrophages. The expression levels of the two osteogenic cytokines BMP‐2 and VEGF were also elevated in the Zn‐coated PEEK group (Figure 3J,K), indicating a probable osteogenic effect in this group.

### 2.4 In Vitro Evaluation of Macrophage Polarization

**Figure 4:**
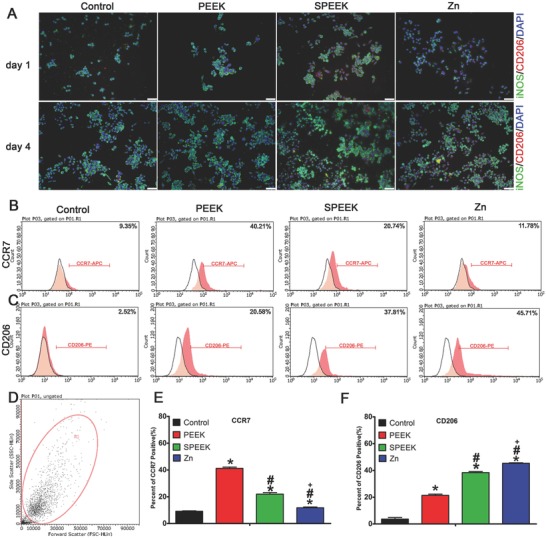
Immunofluorescent staining and surface markers of RAW264.7 cells cultured on samples. A) Immunofluorescent staining of RAW scratched from materials after cultured for 4 d. B,C) Representative dot images of surface markers (CCR7 and CD206) of RAW264.7 analyzed by flow cytometry. D) Representative gate of forward scatter (FSC) and side scatter (SSC). E,F) Percentage of CCR7 or CD206 positive cells respectively. (*, #, and + represent *p* < 0.05 when compared with Control, PEEK, SPEEK, respectively).

Immunofluorescence staining was used to monitor iNOS (green, M1 marker) and CD206 (red, M2 marker) in RAW264.7 macrophages cultured for 1 and 4 d. Clearly, the Zn‐coated PEEK group featured a higher percentage of CD206‐positive cells than other groups, especially at day 4 (**Figure** **4**A). iNOS, however, demonstrated a contrary pattern: more iNOS expression was observed in PEEK group (Figure 4A).

To determine the percentage of M1 or M2 cells, we used flow cytometry to analyze the expression of the surface markers CCR7 and CD206 simultaneously. As shown in Figure 4B,E, the percentage of CCR7‐positive cells decreased from 40.21% in the PEEK group to 11.78% in the Zn‐coated SPEEK group. Furthermore, the percentage of cells expressing the M2 phenotype marker CD206 was higher in the Zn‐coated SPEEK group (45.71%) than in the PEEK (20.58%) and SPEEK (37.81%, Figure 4C,F) groups.

### 2.5 Osteogenic Differentiation Effect of Macrophage‐Conditioned Medium

To evaluate the immunomodulatory osteogenic effect of samples, we prepared macrophage‐conditioned medium. Then, rBMSCs were cultured with the conditioned medium for 7 and 14 d to study their osteogenic differentiation ability.

**Figure 5:**
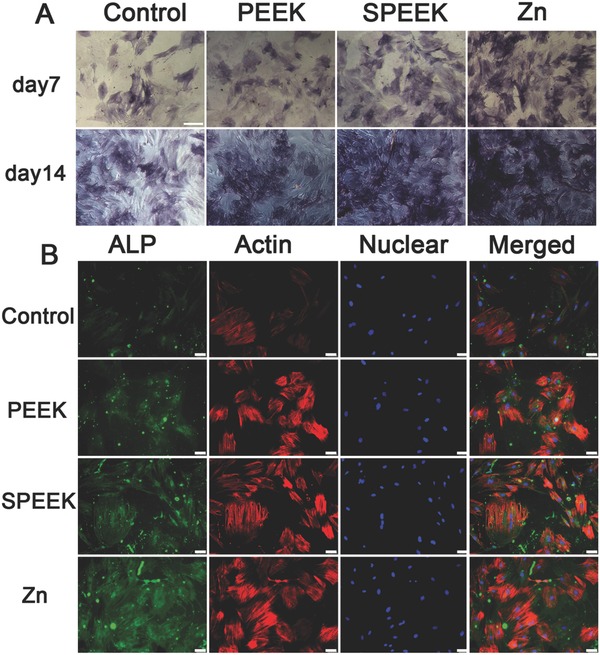
A) ALP staining of rBMSC cultured in conditioned medium for 7 and 14 d. B) ALP immunofluorescent staining of rBMSC in conditioned medium: green (ALP), red (actin), blue (DAPI).

Both alkaline phosphates (ALP) staining and immunofluorescence staining were performed to detected ALP expression in the rBMSCs. **Figure** **5**A reveals that as time progressed, ALP expression increased, and the highest ALP expression was observed in the Zn‐coated SPEEK group, followed by the SPEEK group. Consistent with the ALP staining results, a similar trend was observed in immunofluorescence staining: the highest ALP (green) fluorescence intensity was detected in the Zn‐coated SPEEK group (Figure 5B).

**Figure 6:**
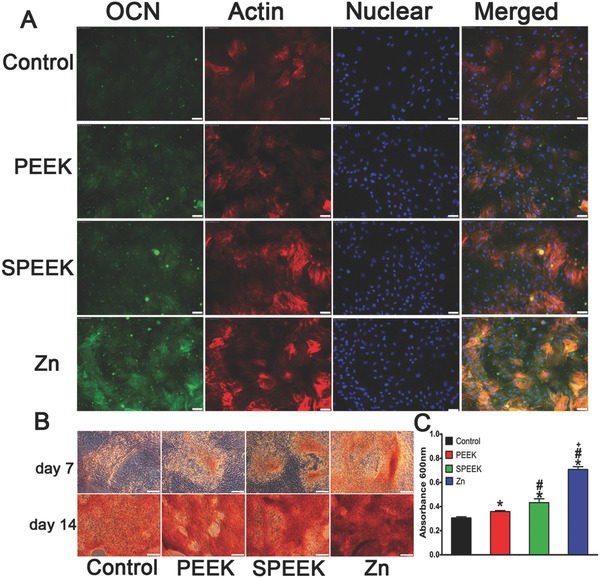
A) OCN immunofluorescent staining of rBMSC in conditioned medium: green (osteocalcin), red(actin), blue (DAPI). B) Alizarin Red staining of rBMSC cultured in conditioned medium for 7 and 14 d. C) Quantitative analysis of Alizarin Red staining. (*, #, and + represent *p* < 0.05 when compared with Control, PEEK, SPEEK, respectively).

We also determined a later osteogenic differentiation protein, osteocalcin (OCN), by immunofluorescence staining at day 14. The results showed that cells in the Zn‐coated SPEEK group expressed more OCN (green) than those in the PEEK or SPEEK group (**Figure** **6**A). To study the mineralization level of rBMSCs in the conditioned medium, we conducted Alizarin red staining at day 7 and 14. More calcified nodules were stained red in the Zn‐coated SPEEK group than the other groups (Figure 6B), and the trend was further confirmed by the quantitative test shown in Figure 6C. RT‐PCR tests were carried out to further determine the expression of osteogenic genes, i.e., OCN, COL‐I, ALP, and RUNX‐2, at day 14. The results are shown in Figure S4 in the Supporting Information. Cells in the Zn‐coated SPEEK group expressed the highest level of the selected genes. No significant difference was found in the expression levels of the four genes between the PEEK and SPEEK groups (Figure S4, Supporting Information). In summary, the conditioned medium of Zn‐coated SPEEK exerted the strongest effects on osteogenic differentiation, which in turn suggested that the macrophages cultured on Zn‐coated SPEEK secreted a series of osteogenic cytokines and showed the best immunomodulatory osteogenic effect.

### 2.6 Results of In Vivo Mouse Air Pouch Model

**Figure 7:**
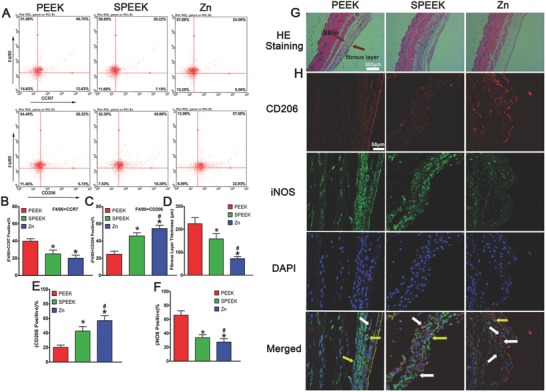
Results of mouse air pouch model. A) Representative dot images of surface markers of cells harvested from air pouches analyzed by flow cytometry. B,C) Percentage of (F4/80+CCR7) or (F4/80+CD206) positive cells respectively. D) Fibrous layer thickness of air pouches' skin. E,F) Percentage of CD206 and iNOS positive cells in the fibrous layer by immunofluorescent staining. G) HE staining of the air pouches' skin. H) Immunofluorescent staining air pouches' skin: red (CD206), green (iNOS), and blue (DAPI). (* and # represent *p* < 0.05 when compared with PEEK, SPEEK, respectively).

Cells in air pouch were collected at day 4 after the operation, and then flow cytometry was used to determine the percentage of M1 and M2 macrophages. Representative dot plots showed that highest proportion of M2 (F4/80+CD206 positive) macrophages was in the Zn‐coated SPEEK group (**Figure** **7**A,C). The percentage of M1 (F4/80+CCR7 positive) macrophages followed the trend PEEK > SPEEK > Zn‐coated SPEEK (Figure 7A,B).

To evaluate the inflammatory level and different phenotypes of the macrophages infiltrating the air pouch skin, we performed hematoxylin and eosin (H&E) and immunofluorescence staining of the skin sections. The thinnest fibrous layer was observed in the Zn‐coated SPEEK group, which indicated a milder inflammatory reaction than the PEEK or SPEEK group (Figure 7D,G). Further immunofluorescence staining of the fibrous layer suggested that a thicker fibrous layer exhibited more M1 macrophages (PEEK group, Figure 7F,H), then a thinner, less inflamed layer, which featured a higher proportion of M2 macrophages (Zn‐coated SPEEK group, Figure 7E,H). Therefore, the mouse air pouch model results were in accordance with the results of the in vitro experiments, and Zn‐coated SPEEK can induce M2 macrophage switching and bring about an anti‐inflammatory environment.

### 2.7 Results of In Vivo Bone Repair Model

**Figure 8:**
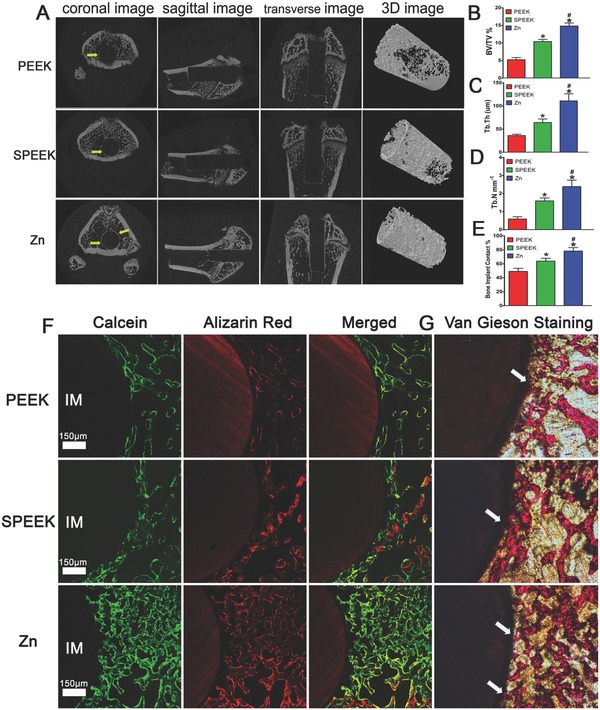
Results of the in vivo bone‐repairing model. A) Coronal, sagittal, transverse, and 3D images of Micro‐CT, yellow arrow indicates new bone. B–D) Quantitative analysis of Micro‐CT data: BV/TV%, Tb.Th, and Tb.N respectively. E) Bone implant contact measured from inset (G). F) Undecalcified sections of sequential polychrome labels for bone: red (Alizarin red), green (Calcein). G) Van Gieson staining of undecalcified sections, white arrow indicates bone implant contact. (* and # represent *p* < 0.05 when compared with PEEK, SPEEK respectively).

The coronal, sagittal, transverse, and 3D microcomputed tomography (micro‐CT) images all supported the conclusion that more new bone formed around Zn‐coated SPEEK (**Figure** **8**A). Further quantitative analysis of the micro‐CT data confirmed that the three indexes reflecting new bone formation (BV/TV, Tb.Th, and Tb.N) in the Zn‐coated SPEEK group were higher than those in the PEEK or SPEEK group (Figure 8B–D).

We prepared undecalcified sections to observe the new bone around the implants through sequential fluorescent labeling and van Gieson staining. Figure 8F reveals that the Zn‐coated SPEEK group featured more stained bones. Van Gieson histological staining was applied to mark the new bone attached to the implants (Figure 8G). Quantitative analysis indicated the highest bone‐implant contact rate was in the Zn‐coated SPEEK group, followed by the SPEEK group (Figure 8E), suggesting that Zn‐coated SPEEK had the strongest bone repair ability.

## 3 Discussion

The field of osteoimmunology has drawn much attention because of the inconsistencies observed between in vitro and in vivo outcomes with direct osteogenic biomaterials. To reach a balance between osteoimmunology and osteointegration, numerous strategies have been applied to modify biomaterials to modulate associated immunological reactions.[[qv: 1a]] Recently, some studies have demonstrated that modified biomaterials can modulate the immune system to improve osteogenesis,[[qv: 8a,15]] which opened another opportunity to enhance the osteointegration of biomaterials. Specifically, it was reported that cytokine‐ or drug‐loaded biomaterials can harness macrophage polarization and generate an osteogenic immune microenvironment.[[qv: 7b,16]] Moreover, some studies suggested that tuning the chemistry and topography of surfaces can manipulate the response of macrophages to biomaterials.^17^ In the present study, we investigated the osteoimmunomodulatory ability of the active ion zinc and its immunomodulatory and osteogenic effects on osteointegration. Zinc, an essential trace element for the human body, participates in numerous metabolic reactions.^18^ The role of zinc in the immune system has been studied for decades.^19^ Zinc deficiency can result in immune cell dysfunction and can increase the risk of inflammation.^20^ In addition, a weakened innate host defense can be observed when zinc homeostasis is disrupted.^21^ Zinc can also enhance the phagocytic ability of macrophages and neutrophils to combat bacteria.^22^ All these findings indicate zinc has an indispensable role in modulating the immune system, especially innate immunity.

Despite the immunomodulatory role of zinc in the immune system, the immunomodulatory and osteogenic effects of zinc remain unclear. In this study, we chose a promising orthopedic material, PEEK, as a substrate. First, we enhanced the biocompatibility of PEEK through sulfonation. Figure 1A shows the morphology of the porous surface, which was beneficial for cell adhesion according to our previous study.^23^ Then, zinc was homogeneously coated on SPEEK through magnetron sputtering (Figure 1C). According to the present results, when macrophages (M0) were seeded on the surface of samples, parts of macrophages would be activated due to the difference between sample surfaces and culture plate (Figure 3). A majority of activated macrophages on PEEK group were activated from M0 to M1 phenotype, though a small part of activated macrophages was M2 phenotype (Figure 4). In contrast, M2 macrophages had major seats on SPEEK and Zn‐coated SPEEK, especially on Zn‐coated SPEEK group. Therefore, both M1 and M2 macrophages could be found on the surface of samples, but the proportion of M1/M2 cells differed among groups. In sum, in vitro immune experiments revealed that Zn‐coated SPEEK can favor macrophage polarization to the M2 phenotype and anti‐inflammatory and osteogenic cytokine secretion (Figures 3 and 4). In fact, some previous reports have already suggested that zinc exerts modulatory effects on the immune system.^14^ Specifically, zinc can induce monocytes to differentiate to macrophages,^24^ and 100 × 10^−6^ m zinc can increase the release of pro‐inflammatory cytokines such as IL‐1, IL‐6, and TNF‐α; however, 1.25 × 10^−6^ m zinc is enough to decrease the expression of pro‐inflammatory cytokines.^25^ The immunomodulatory effect of zinc seems to be dose dependent. Regarding the results of the immersion test (Zn‐coated SPEEK), the concentration of zinc was ≈2.5 × 10^−6^ m (160 µg L^−1^, Figure S3, Supporting Information), which was far lower than 100 × 10^−6^ m. Nevertheless, we should notice that the actual zinc concentration on the material surface that cells interacted with may be higher than 2.5 × 10^−6^ m. Therefore, the optimal concentrations of zinc coated on SPEEK need further investigation. In addition, other studies have indicated that zinc regulated the secretion of cytokines of macrophages through inhibition of the NF‐κB signaling pathway.^26^ It is well known that inhibition of the NF‐κB signaling pathway in macrophages can result in the switch of M0 macrophage to an anti‐inflammatory phenotype, M2.^27^ Further mechanistic analysis verified that zinc can suppress the IκB kinase β (IKKβ) and NF‐κB proteins and then inhibit TNF‐α production.^26^ Therefore, we postulated that Zn‐coated SPEEK exerts immunomodulatory and osteogenic effects by modulating macrophages to an anti‐inflammatory phenotype (M2) and inducing them to secrete some osteogenic cytokines promoting osteogenesis. According to our results, the NF‐κB signaling pathway of macrophages on Zn‐coated SPEEK was inhibited (Figure 3C). Moreover, the Jak‐STAT signaling pathway, which is activated by IL‐4 and induces M2 polarization,^27^ was upregulated, indicating the activity of the M2 phenotype (Figure 3B). Immunofluorescence staining and flow cytometry results validated the higher proportion of M2 macrophages on Zn‐coated SPEEK (Figure 4). As to the higher proportion of the M2 phenotype on SPEEK than on PEEK (Figure 4), reasons may be as follow: it is reported that porous structure can facilitate the attachment and spread of macrophages, then the physical and mechanical signals of porous surface can be translated into biological signals, and subsequently modulate local microenvironment and macrophage polarization.^17^, ^28^ As revealed in Figure 1A, porous structure with pore size about 0.5–1 µm can be observed on the surface of SPEEK, which may provide some biological signals for the polarization of macrophages. In fact, sulfonated PEEK demonstrated a higher proportion of M2 macrophages than PEEK (Figure 4). Recent studies have demonstrated that the M2 cells exhibited an elongated shape compared with M1 cells,^29^ which was in consistent with the present results that the macrophages on SPEEK surface were more elongated than on PEEK surface (Figure 2A). Nevertheless, the higher proportion of M2 macrophages on Zn‐coated SPEEK than SPEEK suggested the immunomodulatory capability of zinc.

Generally, an acute and uncontrollable inflammatory will impair osteogenic differentiation and bone regeneration and ultimately result in implant failure.^30^ In contrast, a mild immune reaction to biomaterials is beneficial for the osteointegration and stability of an implant.^30^ Therefore, to evaluate the osteogenic differentiation ability of the environment generated by Zn‐coated SPEEK, we cultured BMSCs in macrophage‐conditioned medium. The results revealed that the Zn‐coated SPEEK group showed strongest osteogenic differentiation ability (Figures 5 and 6), suggesting that the macrophages cultured on Zn‐coated SPEEK had the highest secretion of osteogenic molecules.

Thus, the previous experiments confirmed that Zn‐coated SPEEK could induce macrophage polarization to the M2 phenotype, thereby promoting osteogenic differentiation in vitro. We next investigated the in vivo inflammatory reaction and macrophage polarization with an air pouch model. A thinner fibrous layer was observed in Zn‐coated SPEEK group, suggesting a comparatively anti‐inflammatory effect (Figure 7G). Fewer inflammatory cells were found in the fibrous layer in the Zn‐coated SPEEK group, which may be due to the decreased expression of colony‐stimulating factors (GM‐CSF, G‐CSF, and M‐CSF, Figure 3A). Furthermore, immunofluorescence images (Figure 7H) revealed that there was a higher percentage of M2 macrophages, which may account for the milder inflammation in the Zn‐coated SPEEK group. When samples were implanted into the air pouches, the foreign body reaction triggered, and neutrophils and macrophages were the first responding cells gathering around the implants. Regarding Zn‐coated SPEEK, a higher proportion of macrophages was induced to the M2 phenotype and thus secreted anti‐inflammatory cytokines (such as IL‐4 and IL‐10). Moreover, M2 macrophages produced less colony‐stimulating factors, which resulted in fewer recruited inflammatory cells and maintenance of the milder inflammatory microenvironment. To verify whether the in vitro osteogenic effects were consistent with the in vivo outcomes, we used a bone defect repair model to evaluate the osteointegration ability of samples. Both micro‐CT and undecalcified section staining (including fluorescence and van Gieson) revealed more new bone around the Zn‐coated SPEEK.

The osteogenic factors, including BMP‐2 and VEGF, may contribute to the favorable osteogenic differentiation and osteointegration of Zn‐coated SPEEK. The macrophages cultured on Zn‐coated SPEEK secreted the highest levels of BMP‐2 and VEGF (Figure 3). BMP‐2, a well‐known osteogenic protein, is an important member of the BMP family.^31^ Enhanced expression of BMP2 will activate the BMP signaling pathway and promote osteogenesis.^32^ In addition, angiogenesis and osteogenesis are two closely connected biological processes.^33^ VEGF is an angiogenesis‐related protein that can bind to the VEGF receptor and activate downstream molecules to increase angiogenesis.^34^ VEGF was thought to have synergistic effects with BMP‐2 on osteogenesis and angiogenesis,^35^ which may be another reason for the superior osteogenesis in the Zn‐coated SPEEK group.

## 4 Conclusion

In summary, we first investigated the osteoimmunomodulatory property of zinc and its effects on osteogenesis by preparing zinc‐coated SPEEK. Both in vitro and in vivo experiments confirmed that Zn‐coated SPEEK can induce macrophage polarization to an anti‐inflammatory phenotype (M2) and then secretion of a set of osteogenic cytokines to improve osteointegration. Our results indicate that zinc shows immunomodulatory and osteogenic effects, which suggested that zinc is a promising and effective additive to develop advanced bone regeneration and immunomodulatory biomaterials.

## 5 Experimental Section

*Fabrication and Modification of Samples*: Samples were cut and polished into 10 mm × 10 mm × 1 mm or 20 mm × 20 mm × 1 mm squares for surface characterization, in vitro cell experiments and in vivo air pouch model. Cylindrical samples of Φ2 × 5 mm^3^ dimensions were employed in rat bone repair model. Prior to sulfonation, all samples were ultrasonically cleaned in acetone, ethanol, and ultrapure water sequentially. To achieve a 3D porous structure, the samples were immersed in sulfuric acid solution (98 wt%) with magnetic stirring for 3 min at room temperature and then were washed with deionized water three times. To remove the remaining sulfuric and acid residues, samples underwent hydrothermal treatment in a 100 mL Teflon‐lined autoclave at 120 °C for 6 h and then were cooled to room temperature. Afterward, zinc was deposited on the sulfonated polyetheretherketone (SPEEK) surface using a magnetron sputtering apparatus (Plasma Technology Ltd., MS400) with a 2 in. Zn metal target (99.99%). Prior to deposition, the deposition chamber was pumped down to ≈10^−4^ Pa, and pure Ar gas (purity, 99.999%) was introduced at 80 sccm. The substrate was rotated at a speed of 10 rpm to obtain a homogeneous zinc coating, and a DC power of 15 W was applied to the target during the sputtering process. Samples prepared with deposition times of 30 s, 1 min, and 2 min were denoted “Zn1”, “Zn2,” and “Zn3,” respectively.

*Surface Characterization of Samples*: The surface morphology of SPEEK and Zn‐coated SPEEK was examined by field‐emission scanning electron microscopy (FE‐SEM, Magellan 400USA). Elemental mapping of Zn‐coated SPEEK was realized by energy‐dispersive X‐ray spectrometry (EDS) using the same machine. The chemical composition, chemical state, and Zn concentrations were studied by X‐ray photoelectron spectroscopy (XPS; Thermo Scientific Escalab 250Xi, US). Argon ion sputtering with an acceleration voltage of 2 kV for 30 s was performed prior to the XPS measurement to obtain a clean surface of each sample. The bonding strength of Zn coating on SPEEK was measured according to the National Standards GB/T 5210‐2006. The wettability of the samples was determined by measuring the contact angle (CA) of a water droplet (10 µL) on the film surface. Digital video imaging was used to process the sessile droplets by a contact angle apparatus (Chengde Dingsheng Testing Machine Co. Ltd, JY‐82A). A charge coupled device (CCD) camera with a space resolution of 1280 × 1024 and a color resolution of 256 gray levels was applied to capture the droplet images.

*Immersion Test*: Samples were immersed in 10 mL of Dulbecco's modified Eagle's medium (DMEM) at 37 °C for 1, 4, 7, or 14 d. At prescribed time, the pH values of and zinc concentrations in the medium were determined by a pH meter (Sartorius, Germany) and an ICP‐MS (Agilent 7500ce, USA), respectively.

*Cell Culture*: Mouse macrophage cells (RAW 264.7) and rat bone marrow mesenchymal stem cells (rBMSCs) were used in this study. Both types of cells were purchased from the Chinese Academy of Science cell bank and cultured in DMEM (HyClone) supplemented with 10% fetal bovine serum (Gibco) and 1% penicillin/streptomycin (Gibco) at 37 °C in a humidified atmosphere of 5% CO_2_. RAW 264.7 cells were passaged by a cell scraper after reaching 80% confluence, while rBMSCs were passaged by trypsinization.

*Cell Adhesion and Morphology on Samples*: Macrophages or rBMSCs (5 × 10^4^) were seeded on samples and cultured for 1 and 4 d. At prescribed time, the cells were dehydrated by ethanol and then dried in hexamethyldisilazane (HMDS). Finally, the samples were sputtered with gold and observed with a scanning electron microscope (Hitachi S‐4800, Japan).

*Cell Proliferation and Viability on Samples*: The CCK‐8 assay and flow cytometry were employed to evaluate the proliferation and viability of macrophages on samples. Cells were seeded on samples at 5 × 10^4^ per well. After culturing for 1 and 4 d, the cells were rinsed with phosphate buffer saline (PBS) and incubated in 10% CCK‐8‐containing medium for 4 h at 37 °C, and then the absorbance of 450 nm was acquired by a spectrophotometer (Bio‐Rad, USA). To determine the cell viability on samples, cells were scratched from the samples and stained with propidium iodide (PI) for 5 min. Finally, a flow cytometer (Millipore, USA) was used to identify the dead cells after culturing for 24 h. RAW 264.7 cells cultured in a 24‐well plate were used as control.

*Gene Expression of Macrophages on Samples*: A microarray was used to detect the gene expression profile of macrophages on samples. Briefly, RAW 264.7 cells were seeded on samples (1 × 10^5^ per well) and cultured for 4 d. Then, cell RNA was harvested by TRIzol reagent, and the whole gene expression was examined at the Beijing Genomics Institute (BGI, China). Fold changes in the expression of selected genes were exhibited by a heat map, and pathway enrichment was evaluated by KEGG pathway analysis.

After incubation on samples for 4 d, the culture medium of macrophages was collected and centrifuged. Then, ELISA kits (Anogen, Canada) were used to examine the concentrations of TNF‐α, IL‐4, IL‐6, and IL‐10 in the supernatants according the manufacturer's instructions.

RT‐PCR was also performed to investigate the expression levels of the M1 macrophage marker CCR7, the M2 macrophage marker CD206, and the osteogenic factors BMP‐2 and VEGF. Cells were cultured on samples for 4 d, and then RNA was extracted by TRIzol reagent. Complementary DNA (cDNA) was synthesized from 2 µg of total RNA using reverse transcriptase M‐MLV (Takara). Quantitative gene analysis was performed using RT‐PCR with SYBR Premix Ex Taq (Takara) (SYBR = *N*′,*N*′‐dimethyl‐*N*‐[4‐[(E)‐(3‐methyl‐1,3‐benzothiazol‐2‐ylidene)methyl]‐1‐phenylquinolin‐1‐ium‐2‐yl]‐*N*‐propylpropane‐1,3‐diamine). The primers used in this section are shown in Table S2 (Supporting Information) with β‐actin as a housekeeping gene. RAW 264.7 cells cultured in 12‐well plate were used as control.

*In Vitro Polarization of Macrophages Cultured on Samples*: Fluorescence staining was carried out to evaluate the expression levels of iNOS (green, M1 marker) and CD206 (red, M2 marker). After incubation on samples for 1 and 4 d, macrophages were scratched and seeded in a 12‐well plate for 30 min for reattachment. Subsequently, the cells were fixed in 4% paraformaldehyde, permeabilized by 0.1% Triton‐X for 30 min, blocked using 1% bovine serum albumin (BSA) for 1 h and incubated with primary antibodies for iNOS (1:50, Novus Biologicals) and CD206 (1:50, Abcam) overnight at 4 °C. Secondary antibody donkey anti‐rabbit Alexa Fluor 488 (1:200, Abcam) and donkey anti‐mouse Alexa Fluor 594 (1:200, Abcam) was applied to combine with the primary antibody for 2 h. Finally, the nuclei were stained blue with 4',6‐diamidino‐2‐phenylindole, pihydrochloride (DAPI) and observed with a fluorescence microscope (Leica).

A flow cytometer was further used to obtain the proportion of M1 and M2 macrophages. The antibodies used in this test were purchased from eBioscience. Briefly, cells were scratched into Eppendorf (EP) tubes after culturing on samples for 4 d. Cells were centrifuged, rinsed with 1% BSA to block nonspecific antigens for 30 min, and then stained by allophycocyanin (APC)‐conjugated CCR7 and phycoerythrin (PE)‐conjugated CD206 for 1 h in the dark. Meanwhile, fluorescein isothiocyanate (FITC)‐conjugated rat IgG2a,κ, APC‐conjugated rat IgG2a,κ, and PE‐conjugated rat IgG2a,κ were used as isotype controls. Finally, 100 µL cell suspensions were added to a 96‐well plate and detected by a Guava flow cytometer (Millipore, USA). Data were also analyzed by Guava software 3.1.1, and RAW 264.7 cells cultured in 12‐well plate were used as a control.

*Osteogenic Differentiation Evaluation of Macrophage‐Conditioned Medium*: Conditioned medium was prepared by culturing macrophages on different samples for 4 d, collecting the supernatants of the culture medium and mixing them with DMEM at a 1:2 ratio. rBMSCs was cultured on a 24‐well plate at a density of 1 × 10^4^ cells per well with DMEM, and after incubation for 12 h, the medium was replaced by conditioned medium for further incubation. At days 7 and 14, the cells were fixed and stained with ALP or Alizarin red dye according to the ALP staining kit (Beyotime) or Alizarin red dye (Cyagen) instructions, respectively. Quantitative analysis of Alizarin red staining was achieved by dissolving the deposited calcium with 10% cetylpyridinium chloride and detecting the optical density (OD) values of the solution at 600 nm.

Two representative proteins (ALP and OCN) indicating osteogenic differentiation were determined by fluorescence staining. After they were cultured in conditioned medium for specific times, the cells were fixed in 4% paraformaldehyde, permeabilized with 0.1% Triton‐X for 30 min, blocked with 1% BSA for 1 h, and incubated with primary antibodies overnight at 4 °C. Subsequently, secondary antibodies were applied to attach to the primary antibodies. Finally, the cytoskeleton and nuclei were stained with phalloidin and DAPI, respectively. A fluorescence microscope (Leica) was used to acquire representative images, and rBMSCs cultured in DMEM were used as a control.

*In Vivo Immunomodulatory Evaluation of Samples*: The animal experiments were approved by the Animal Care Committee of Shanghai Jiao Tong University Affiliated Sixth People's Hospital, and all the operations on animals were in accordance with the guidelines established by the Administration of Affair Concerning Laboratory Animals for Shanghai Jiao Tong University and the National Institutes of Health Guide for Care and Use of Laboratory Animals (GB14925‐2010). An air pouch was first developed on the back of C57BL/6 mice by injecting sterile air subcutaneously according to the previous study. Then, the mice were anaesthetized via intraperitoneal injection of pentobarbital and shaved and sterilized the skin of the air pouch. An incision was made on the middle of the pouch, one sample (10 mm squares) was inserted into the pouch, and the incision was closed gently. All the surgical procedures were performed in an aseptic manner. Four days after the surgery, the mouse was sacrificed. The inflammatory cells in the air pouch were harvested by washing the cavity with 2 mL of PBS. Then, a flow cytometer was used to determine the proportion of M1 and M2 macrophages. During the tests, the macrophages were stained with FITC‐conjugated anti‐mouse F4/80. Other procedures were similar to in vitro flow cytometer test mentioned above.

The skin covering the implants were harvested and fixed in paraformaldehyde. Further tissue sections (≈5 µm) were made after embedding in paraffin. Then, the sections were stained with hematoxylin and eosin (H&E) to evaluate the inflammatory reaction of the skin. Immunofluorescence staining was used to quantify the percentage of different phenotypes of macrophages in the fibrous layer. The staining procedures were carried out using the same antibodies as in vitro macrophages immunofluorescence staining according to the manufacturer's instructions.

To investigate the in vivo bone regeneration of samples, a rat bone repair model was built. Cylindrical implants (2 mm diameter, 5 mm long) were inserted parallel to the long axis in the femurs (one implant per femur) of rats for 8 weeks. At 4 and 6 weeks after the surgery, calcein and Alizarin red were injected intraperitoneally to mark the new bone. When the rats were sacrificed by an overdose of pentobarbitone sodium at week 8, the femurs containing the implants were harvested and subjected to micro‐CT scanning. Then, the femurs were embedded in polymethylmethacrylate (PMMA), and undecalcified sections were acquired using a Leica diamond saw (Leica SP1600). A confocal laser scanning microscope (Leica CLSM) was used to observe polychrome‐labeled bone. Finally, the sections were stained with van Gieson dye and observed by an optical microscope (Leica) to display the bone implant contact.

*Statistical Analysis*: Count data were presented as the mean ± standard deviation. Differences among groups were analyzed with one‐way ANOVA followed by the Student‐Newman‐Keuls (SNK) test using SPSS20.0. *P* < 0.05 was considered a significant difference.

## Conflict of Interest

The authors declare no conflict of interest.

## Supporting information

Supplementary
