# Supplementary material for: Zinc‐Modified Sulfonated Polyetheretherketone Surface with Immunomodulatory Function for Guiding Cell Fate and Bone Regeneration
Source: Adv Sci (Weinh). 2018 Aug 7;5(10):1800749. doi: 10.1002/advs.201800749 (PMC6193167; doi:10.1002/advs.201800749)
Supplement: Supplementary file 1 — Supplementary [file ADVS-5-1800749-s001.pdf]

Copyright WILEY-VCH Verlag GmbH & Co. KGaA, 69469 Weinheim, Germany, 2018.

ADVANCED SCIENCE
Open Access

Supporting Information

for *Adv. Sci.*, DOI: 10.1002/advs.201800749

Zinc-Modified Sulfonated Polyetheretherketone Surface with Immunomodulatory Function for Guiding Cell Fate and Bone Regeneration

Wei Liu, Jinhua Li, Mengqi Cheng, Qiaojie Wang, Kelvin W. K. Yeung, Paul K. Chu,* and Xianlong Zhang**

Supporting Information

Zinc-Modified Sulfonated Polyetheretherketone Surface with Immunomodulatory Function for Guiding Cell Fate and Bone Regeneration

Wei Liu, Jinhua Li, Mengqi Cheng, Qiaojie Wang, Kelvin W. K. Yeung, Paul K. Chu*, and Xianlong Zhang**

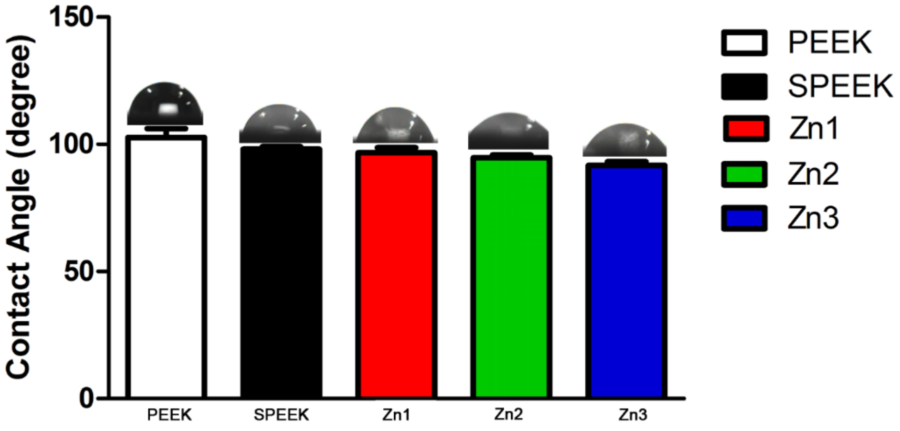

Figure S1. Measurement results of water contact angle of the samples.

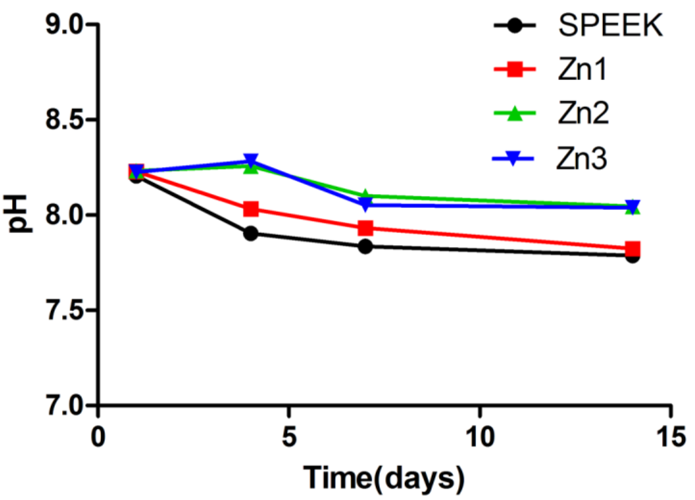

Figure S2. pH values of different samples immersed in DMEM medium for different times.

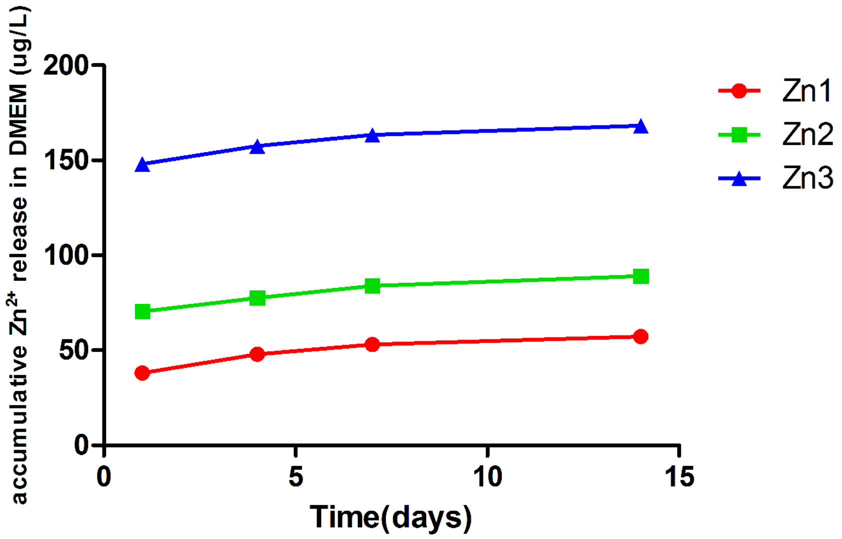

Figure S3. Zn concentrations of different samples immersed in DMEM medium for different time.

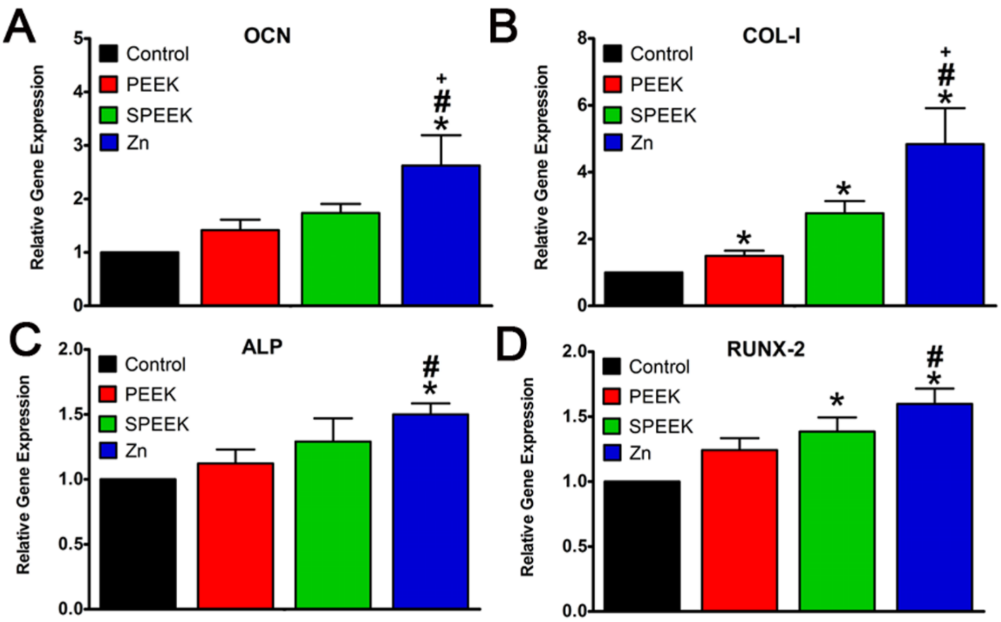

Figure S4. Osteogenic related genes expression of rBMSC cultured in conditioned medium by RT-PCR at day 14. (*, # and + represent $p < 0.05$ when compared with Control, PEEK, SPEEK respectively).

Table S1. The Zn content and bonding strength of Zn coating for Zn-coated SPEEK.

Sample	Zn1-SPEEK	Zn2-SPEEK	Zn3-SPEEK
Zn content	3.57%	6.73%	15.97%
Bonding strength	5.2 MPa	5.4 MPa	5.5 MPa

Table S2. Primers used in the RT-PCR test of RAW264.7 cells.

Gene	Primers Sequence (F, forward; R, reverse; 5'−3')	Product Size (bp)
CD206	F: TACTTGGACGGATAGATGGAGG<br>R: CATAGAAAGGAATCCACGCAGT	287
CCR7	F: GGTGGCTCTCCTTGTCATTTTC<br>R: AGGTTGAGCAGGTAGGTATCCG	264
VEGF	F: AGGAGTACCCCGACGAGATAGA<br>R: CACATCTGCTGTGCTGTAGGAA	198
BMP-2	F: AACGAGAAAAGCGTCAAGCC<br>R: AGGTGCCACGATCCAGTCAT	119
β-actin	F: GTGACGTTGACATCCGTAAAGA<br>R: GTAACAGTCCGCCTAGAAGCAC	287

Table S3. Primers used in the RT-PCR of rBMSC cells.

Gene	Primers Sequence (F, forward; R, reverse; 5'−3')	Product Size (bp)
ALP	F: ATGCTCAGGACAGGATCAAA<br>R: CGGGACATAAGCGAGTTTCT	78
COL-I	F: AGCTCGATACACAATGGCCT<br>R: CCTATGACTTCTGCGTCTGG	209
RUNX-2	F: ATCATTCAGTGACACCACCA<br>R: GTAGGGGCTAAAGGCAAAAG	141
OCN	F: CAGACAAGTCCCACACAGCA<br>R: CCAGCAGAGTGAGCAGAGAG	85
β-actin	F: CCTCTATGACAACACAGT<br>R: AGCCACCAATCCACACAG	155
